# Systemic nature of spinal muscular atrophy revealed by studying insurance claims

**DOI:** 10.1371/journal.pone.0213680

**Published:** 2019-03-14

**Authors:** Scott L. Lipnick, Denis M. Agniel, Rahul Aggarwal, Nina R. Makhortova, Samuel G. Finlayson, Alexandra Brocato, Nathan Palmer, Basil T. Darras, Isaac Kohane, Lee L. Rubin

**Affiliations:** 1 Department of Stem Cell and Regenerative Biology, Harvard University, Cambridge, Massachusetts, United States of America; 2 Department of Biomedical Informatics, Harvard Medical School, Boston, Massachusetts, United States of America; 3 Center for Assessment Technology & Continuous Health (CATCH), Massachusetts General Hospital, Boston, Massachusetts, United States of America; 4 Boston Children’s Hospital, Boston, Massachusetts, United States of America; 5 Harvard Stem Cell Institute, Cambridge, Massachusetts, United States of America; Providence Care Hospital, CANADA

## Abstract

**Objective:**

We investigated the presence of non-neuromuscular phenotypes in patients affected by Spinal Muscular Atrophy (SMA), a disorder caused by a mutation in the *Survival of Motor Neuron (SMN)* gene, and whether these phenotypes may be clinically detectable prior to clinical signs of neuromuscular degeneration and therefore independent of muscle weakness.

**Methods:**

We utilized a de-identified database of insurance claims to explore the health of 1,038 SMA patients compared to controls. Two analyses were performed: (1) claims from the entire insurance coverage window; and (2) for SMA patients, claims prior to diagnosis of any neuromuscular disease or evidence of major neuromuscular degeneration to increase the chance that phenotypes could be attributed directly to reduced SMN levels. Logistic regression was used to determine whether phenotypes were diagnosed at significantly different rates between SMA patients and controls and to obtain covariate-adjusted odds ratios.

**Results:**

Results from the entire coverage window revealed a broad spectrum of phenotypes that are differentially diagnosed in SMA subjects compared to controls. Moreover, data from SMA patients prior to their first clinical signs of neuromuscular degeneration revealed numerous non-neuromuscular phenotypes including defects within the cardiovascular, gastrointestinal, metabolic, reproductive, and skeletal systems. Furthermore, our data provide evidence of a potential ordering of disease progression beginning with these non-neuromuscular phenotypes.

**Conclusions:**

Our data point to a direct relationship between early, detectable non-neuromuscular symptoms and SMN deficiency. Our findings are particularly important for evaluating the efficacy of SMN-increasing therapies for SMA, comparing the effectiveness of local versus systemically delivered therapeutics, and determining the optimal therapeutic treatment window prior to irreversible neuromuscular damage.

## Introduction

Spinal Muscular Atrophy (SMA) is a rare autosomal recessive disorder caused by a pair of missing or defective *Survival of Motor Neuron 1* (*SMN1*) genes [1]. A modifier gene, *SMN2*, also produces SMN protein, but, due to a point mutation, the yield is only a fraction of what is produced by *SMN1* [2]. As the name of the SMN protein implies, the SMA pathology is widely thought to be attributed to the highly selective degeneration of motor neurons. As such, disease severity is typically described by age of detectable changes in motor symptoms and best motor performance achieved [3]. This simplistic representation of SMA is not unlike that of other neurological disorders in which each disease is defined and studied primarily in the context of the near-exclusive involvement of a specific subtype of central nervous system (CNS) neurons.

However, growing evidence demonstrates that SMN has a broader role across cell types and physiological systems, such as cardiovascular, intestinal and skeletal, indicating that SMA may be a multi-system disorder [4–9]. This builds on recent studies that showed the *SMN1/2* genes are expressed in every human [10] and mouse [11] tissue, and are required for the viability of all eukaryotic cells [12–14]. Validation of the functional importance of SMN using SMA mouse models has proved challenging, as many of the most commonly used models, especially the delta7 mouse [15], generally die within two weeks of birth. Furthermore, these mice have severe motor defects that when combined with their limited lifespan make it challenging to study multiple tissue involvement or establish a clear path of disease progression [16]. Even so, the use of these models has been extremely valuable and led directly to the development of Spinraza, the only FDA approved therapeutic for SMA. Even though Spinraza is delivered intrathecally, clinical measurements made in young children have provided evidence for detectable concentrations in the kidney and liver [17]. Nonetheless, it is highly likely that Sprinraza’s main direct benefits are limited to cells within the spinal cord [18]. Importantly, data obtained using the SMA mouse model show that treatments like Spinraza that solely target the CNS fail to completely reduce overall disease severity [19–21], while ones delivered peripherally are more successful at prolonging survival [22, 23]. Moreover, therapies delivered after detectable neuromuscular degeneration has occurred have a limited capacity to alter disease progression [23, 24], emphasizing the need for early intervention. Taken together, there is reason to believe that a more complete system-wide physiological understanding of SMA and a greater understanding of its temporal progression will be valuable [24, 25].

In the present work, we show, for the first time, that an unbiased analysis of available clinical data yields evidence that patients affected by SMA have multiple tissue defects, some of which present prior to detectable neuromuscular symptoms and are not likely to be attributable to neuromuscular deterioration. These aspects of SMA include, but are not limited to, cardiovascular, gastrointestinal, metabolic, reproductive, and skeletal defects. To accomplish our analyses, we utilized methods employed in previous studies that mined large medical datasets to develop risk-prediction models and characterize disease progression [26–28]. Our results have major implications as they highlight the need for broader systemic monitoring of SMA progression and provide a framework for comparative effectiveness studies between locally delivered therapeutics, such as Spinraza, and those delivered systemically, some of which may be nearing clinical approval. Finally, while newborn screening for SMA will support early intervention for the severe patients (Types I/II), later stage patients (Types III/IV) may benefit from improved methods of tracking disease progression including during the stage when they are still pre-symptomatic for neuromuscular weakness.

## Materials and methods

The Harvard Medical School Institutional Review Board approved this research.

### Data

We utilized a de-identified administrative database from Aetna Inc. representing 63,444,784 unique members during a period extending from January 1, 2008 through October 1, 2015. For each individual, we extracted gender, year of birth, enrollment duration, and diagnoses in the form of International Classification of Diseases, 9^th^ Revision (ICD9), Clinical Modification codes.

### Subject/Control selection

Using a selection approach similar to one used to evaluate the economic burden of SMA [29], we first selected all individuals with at least 2 ICD9 codes for SMA on different visits. Next, to avoid inclusion of subjects that might have been misdiagnosed with SMA, such as those affected by muscular dystrophy (MD) and myoneural disorders, we required the final neuromuscular disease diagnosis on record to be SMA. Additionally, after reviewing each of the selected individuals, we found that a large number of women undergoing prenatal genetic testing or care for pregnancies with a high risk for SMA received SMA diagnoses for billing purposes. To overcome this, we excluded any subject with pregnancy codes or codes related to pregnancy complications as detailed in S1 Table.

**Fig 1:**
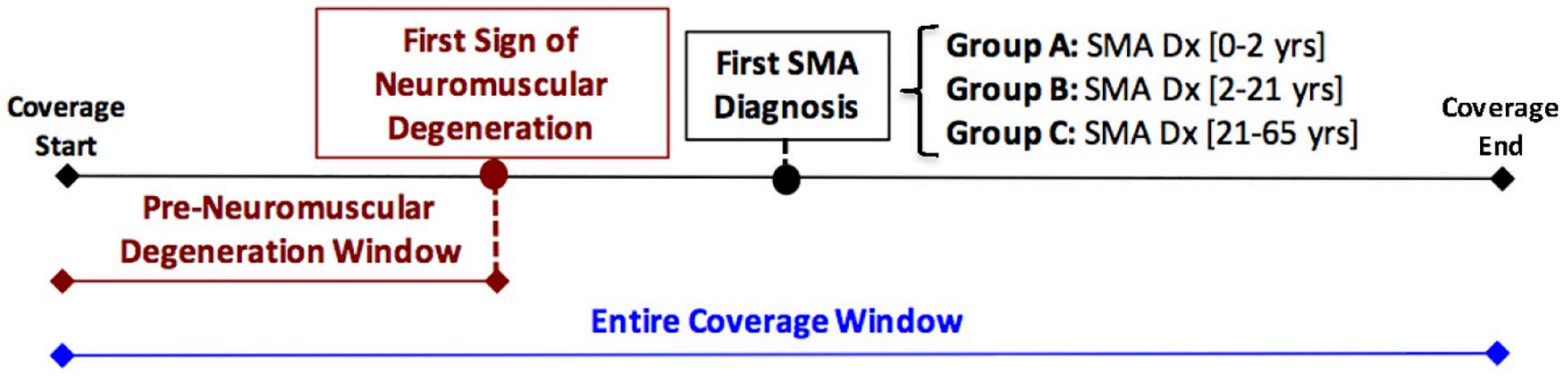
Patient selection and grouping schematic. SMA patients were identified based on having at least 2 SMA diagnosis codes in their insurance coverage window. Additionally, their diagnosis must have been associated with a final diagnosis of SMA as opposed to another similarly treated neuromuscular disorder. Using these cohorts, two analyses were performed. The first, included the entire coverage window (Entire Coverage Window Analysis) with a 6-month minimum observation window. The second, Pre-Neuromuscular Degeneration Analysis, only included data prior to the first neuromuscular disease diagnosis or evidence of major neuromuscular degeneration with a minimum observation window of 6 months. The SMA cohort was split into three groups by age at first SMA diagnosis on record.

Confirmed SMA patients were then stratified into three subcategories based on age at first SMA diagnosis on record [1]: Group A represented likely SMA types I/II patients having diagnoses from birth until 2 years of age; Group B represented likely SMA types III patients with diagnoses from 2 until 21 years of age; and Group C represented likely SMA type IV patients with diagnoses between 21 and 65 years of age. Control populations were selected to match the range of ages at insurance enrollment for each SMA Group. As such, control Groups may share individuals given that there is an overlap in the age at insurance enrollment for the SMA Groups since their stratification is based on age at first SMA diagnosis. For each patient/control Group, two different analyses were performed as shown in Fig 1. The first analysis utilized the entire coverage window of each individual, whereas the second included, for SMA cases, only data prior to the first diagnosis of neuromuscular disease (SMA, MD, etc.) or evidence of major neuromuscular degeneration defined by the presence of certain ICD9 codes (S1 Table). In this manuscript, this time-point is referred to as the first sign of neuromuscular degeneration, with the time prior to that being their pre-neuromuscular degeneration window. Finally, in order to have sufficient depth of health records, we required each subject to have at least 6 months of coverage during the analysis window. For the pre-neuromuscular degeneration analysis, the required six-months prior to their first sign of neuromuscular degeneration supports exclusion of patients that potentially had an SMA diagnosis prior to their coverage.

### Phenotypic differences

To reduce the number of variables tested, we first converted ICD9 codes to phenome-wide-association (PheWAS) diagnosis groups [30]. This allowed us to aggregate similar ICD9 codes that, for the purpose of this study, can be considered diagnoses of equivalent conditions. In order to test whether PheWAS groups were diagnosed at significantly different rates between SMA and control groups and to obtain covariate-adjusted odds ratios (OR), we regressed an indicator of the PheWAS group onto an indicator of SMA diagnosis using logistic regression via the glm() function in R-3.3.3 [31] with gender, age at insurance enrollment, and enrollment months during the analysis window as covariates. Only phenotypes with prevalence of at least 1% in the SMA Group or control population were evaluated. The false discovery rate (FDR) was controlled at 5% using the Benjamini-Hochberg procedure [32] to adjust p-values.

### Temporal trajectories of the onset of categorized phenotypes

Next, for only the pre-neuromuscular degeneration analysis window, we took all phenotypes with adjusted p-values less than 0.05 and grouped them by physiological system to evaluate broader categories of disease-associated dysfunctions and repeated the logistic regression analyses where now the outcome was the binary indicator of any phenotype covered by the specified physiological system. To characterize a potential timeline of disease progression, we computed the median time between first diagnosis of any ICD9 code covered by each specified physiological system and a patient’s first sign of neuromuscular degeneration. The goal was to ascertain whether there is a temporal relationship between the onsets of various non-neuromuscular defects and that of neuromuscular dysfunction. To eliminate the possibility that these timelines could result from a technical artifact due to variability in the length of a subject’s insurance coverage, we calculated the R^2^ coefficient of determination between first diagnoses of specific defects for all SMA cases with their insurance enrollment duration.

## Results

### Patient/Control cohorts

For the analysis utilizing the entire coverage window period, we identified 1,038 SMA cases that fit our inclusion and exclusion criteria (79 in Group A [average age = 0.34yrs]; 351 in Group B [9.44yrs]; 608 in Group C [44.17yrs]); 39,214,424 controls (1,536,458 for Group A [0.35yrs]; 10,709,155 for Group B [9.48yrs]; and 26,968,811 for Group C [39.72yrs]). For the analysis of data only in the pre-neuromuscular degeneration window, our cohort included 475 SMA patients (31 in Group A [0.16yrs]; 107 in Group B [8.51yrs]; 337 in Group C [44.74yrs]) and 38,822,115 controls (1,536,458 for Group A [0.35yrs]; 10,709,155 for Group B [9.48yrs]; 26,576,502 for Group C [39.35yrs]). Complete details on each cohort including total population, gender, age at insurance enrollment, mean enrollment duration, and a measure of medical utilization in the form of days with ICD9 codes per six-month period are presented in Table 1.

**Table 1:** Patient cohort details.

	Disease Status	Population	% Female	Mean Age at Enrollment (yrs)	Mean Enrollment (mos)	Age at First SMA Diagnosis (yrs)	Days with ICD codes per 6mo
**Analysis or Entire Coverage Window**							
Group A:	sma	79	51.90%	0.34	30.99	0.67	18.68
	ctrl	1,536,458	48.65%	0.35	35.06	--	4.54
	p-value	--	56.79%	9.E-01	9.E-02	--	7.E-22
Group B:	sma	351	47.58%	9.44	47.66	10.01	14.17
	ctrl	10,709,155	49.29%	9.48	38.99	--	2.83
	p-value	--	52.24%	9.E-01	1.E-07	--	1.E-62
Group C:	sma	608	45.89%	44.17	52.99	45.31	10.19
	ctrl	26,968,811	54.21%	39.72	38.06	--	3.76
	p-value	--	0.00%	1.E-17	3.E-32	--	2.E-70
**Analysis of Pre-Neuromuscular Degeneration Window**							
Group A:	sma	31	48.39%	0.16	13.94	0.87	8.23
	ctrl	1,536,458	48.65%	0.35	35.06	--	4.54
	p-value	--	97.69%	9.E-03	5.E-25	--	5.E-04
Group B:	sma	107	51.40%	8.51	26.80	10.18	4.68
	ctrl	10,709,155	49.29%	9.48	38.99	--	2.83
	p-value	--	66.40%	1.E-01	8.E-11	--	9.E-04
Group C:	sma	337	42.14%	44.74	30.85	46.64	4.90
	ctrl	26,576,502	54.21%	39.35	38.09	--	3.74
	p-value	--	0.00%	1.E-14	1.E-10	--	1.E-04

Characteristics including population, gender, age at enrollment, mean enrollment months, age at first SMA diagnosis, and medical utilization in the form of days with ICD9 codes per 6 months are presented. Controls, selected as all subjects with no SMA codes matched by age at enrollment range, are presented. Cohorts are split into three groups (Group A: SMA diagnosis of > = 0 & <2yrs; Group B: SMA diagnosis of > = 2 & <21yrs; and Group C: SMA diagnosis of > = 21 & < = 65yrs).

### Identification of non-neuromuscular phenotypes in SMA patients

**Fig 2:**
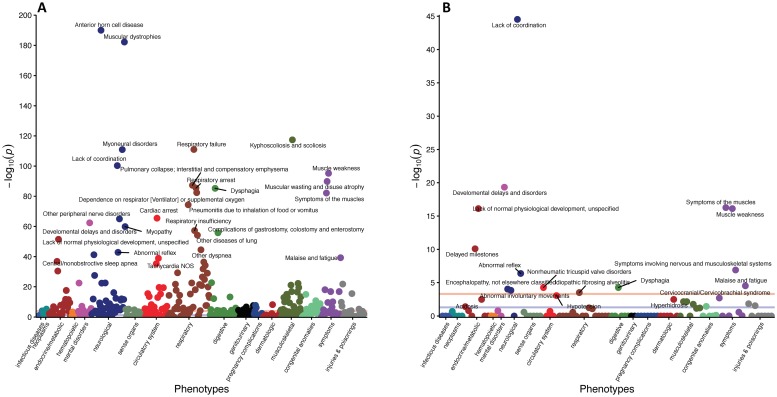
Manhattan plots of phenotypes with increased prevalence in SMA for Group A. (A) Phenotypes with increased prevalence in SMA patients compared to controls throughout their entire coverage window. Only selected phenotypes with p-value<4e-25 are displayed. (B) Phenotypes with increased prevalence in SMA patients compared to controls from only data prior to the first sign of neuromuscular degeneration. Only phenotypes with p-value<0.05 are displayed. For both plots, the purple line represents an adjusted p-value of 5x10^(-2)^ and the peach line represents an adjusted p-value of 5x10^(-4)^. Completes lists of phenotypes are presented in the supplemental tables.

**Fig 3:**
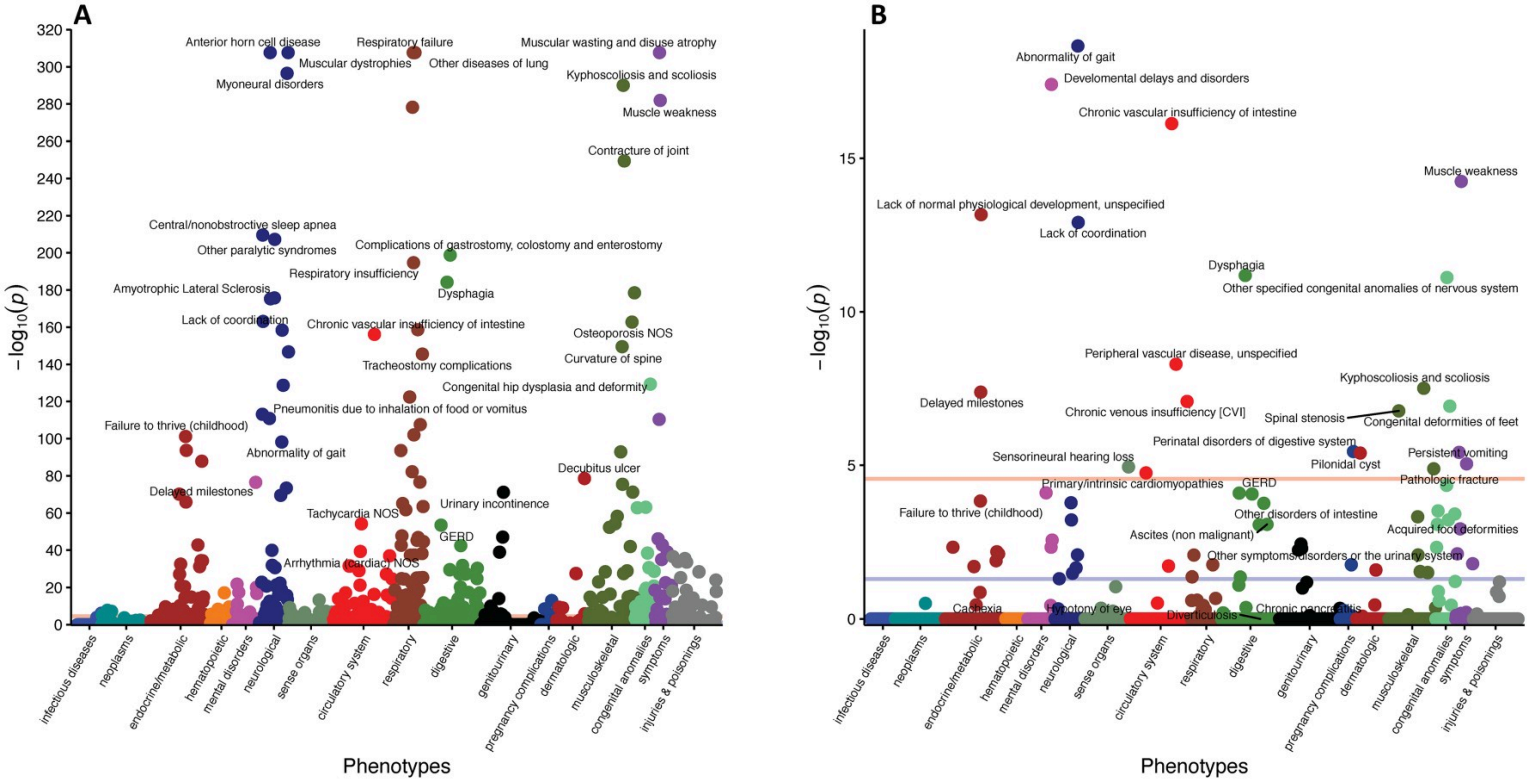
Manhattan plots of phenotypes with increased prevalence in SMA for Group B. (A) shows phenotypes with increased prevalence in SMA patients compared to controls throughout their entire coverage window. Only selected phenotypes with p-value<1e-50 are displayed. (B) Phenotypes with increased prevalence in SMA patients compared to controls from only data prior to their first sign of neuromuscular degeneration. Only phenotypes with p-value<0.005 are displayed. For both plots, the purple line represents an adjusted p-value of 5x10^(-2)^ and the peach line represents an adjusted p-value of 5x10^(-4)^. Completes lists of phenotypes are presented in the supplemental tables.

**Fig 4:**
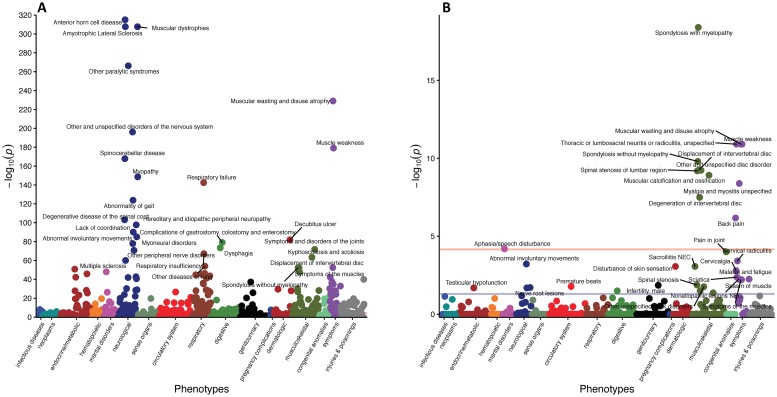
Manhattan plots of phenotypes with increased prevalence in SMA for Group C. (A) shows phenotypes with increased prevalence in SMA patients compared to controls throughout their entire coverage window. Only selected phenotypes with p-value<1e-47 are displayed. (B) Phenotypes with increased prevalence in SMA patients compared to controls from only data prior to their first sign of neuromuscular degeneration. Only phenotypes with p-value<0.021 are displayed. For both plots, the purple line represents an adjusted p-value of 5x10^(-2)^ and the peach line represents an adjusted p-value of 5x10^(-4)^. Completes lists of phenotypes are presented in the supplemental tables.

Differential diagnoses or phenotypes, in the form of PheWAS groups, from the entire coverage window of SMA patients compared to controls for Groups A, B, and C are presented in S2–S4 Tables. These data reveal a host of non-neuromuscular phenotypes, as well as more traditional hallmarks of disease progression including muscular, respiratory, feeding, and mobility complications. Adjusted p-values for each phenotype, organized by primary associated physiological system, are presented using Manhattan plots in Figs 2A, 3A and 4A. Notably, non-traditional aspects of the disease that are revealed include, but are not limited to, cardiovascular (peripheral vascular disease, tachycardia, chronic vascular insufficiencies of the intestine), metabolic (hypoglycemia, hypopotassemia (hypokalemia)), and sensorial (chronic pain syndrome, pancreatitis) defects.

Since this analysis included features detected post SMA-diagnosis, the determination of whether a phenotype is an independent feature of SMA, directly attributable to reduced SMN levels, or simply a consequence of neuromuscular dysfunction is unclear. Therefore, we repeated the analysis, but limited the data to the period prior to clinically detectable neuromuscular degeneration. The analysis of data collected in that period yielded a total of 17, 67, and 35 differential phenotypes for Groups A, B, and C are presented in S5–S7 Tables, respectively. Visualization of the relative significance of each phenotype is presented using Manhattan plots (Figs 2B, 3B and 4B), alongside data from the entire coverage window. Selected non-neuromuscular phenotypes of particular interest representing cardiovascular, gastrointestinal, and male reproductive defects are also provided in Table 2. These data demonstrate the broad impact of reduced SMN outside of the CNS and in advance of neuromuscular degeneration. While it is possible that some of the phenotypes could be consequences of the earliest stages of neuromuscular dysfunction, others, such as defects in the cardiovascular and male reproductive systems, are more definitively independent.

**Table 2:** Selected phenotypes occurring prior to overt neuromuscular involvement.

SMA Group	PheWAS Code	Phenotype	p-adjusted	OR	Group
**Group A**	395.3	Nonrheumatic tricuspid valve disorders	5.4E-05	42.23	Cardiovascular
	532	Dysphagia	5.4E-05	15.62	Gastrointestinal
**Group B**	441.2	Chronic vascular insufficiency of intestine	7.5E-17	683.32	Cardiovascular
	443.9	Peripheral vascular disease, unspecified	5.1E-09	122.27	Cardiovascular
	456	Chronic venous insufficiency [CVI]	8.3E-08	85.70	Cardiovascular
	425.1	Primary/intrinsic cardiomyopathies	1.8E-05	40.77	Cardiovascular
	747.13	Congenital anomalies of great vessels	8.4E-04	9.07	Cardiovascular
	747.11	Cardiac shunt/ heart septal defect	4.7E-03	5.83	Cardiovascular
	532	Dysphagia	6.5E-12	14.46	Gastrointestinal
	656.6	Perinatal disorders of digestive system	3.6E-06	30.10	Gastrointestinal
	789.1	Persistent vomiting	9.0E-06	15.47	Gastrointestinal
	530.11	GERD	8.1E-05	4.09	Gastrointestinal
	561.2	Flatulence	1.1E-04	9.03	Gastrointestinal
	569	Other disorders of intestine	1.7E-04	15.37	Gastrointestinal
	572	Ascites (non-malignant)	8.4E-04	21.17	Gastrointestinal
	560.4	Other intestinal obstruction	8.7E-04	20.89	Gastrointestinal
	563	Constipation	1.3E-02	2.84	Gastrointestinal
	530.14	Reflux esophagitis	4.3E-02	5.80	Gastrointestinal
**Group C**	427.6	Premature beats	1.7E-02	3.32	Cardiovascular
	563	Constipation	5.7E-03	2.29	Gastrointestinal
	532	Dysphagia	3.2E-02	2.35	Gastrointestinal
	257.1	Testicular hypofunction	2.1E-02	2.37	Male Reproductive
	609.1	Infertility, male	1.4E-02	5.10	Male Reproductive

Non-neuromuscular phenotypes detected prior to first sign of neuromuscular degeneration from each group representing cardiovascular, gastrointestinal, and male reproductive defects are presented with adjusted p-values, odds ratios, and prevalence for each cohort.

Despite having had a limited length of time prior to their first sign of neuromuscular degeneration, children from Group A showed evidence of numerous non-neuromuscular phenotypes. These phenotypes included: cardiovascular (non-rheumatic tricuspid valve disorders: 6.5% in SMA, OR = 42, adjusted p-value<0.001); gastrointestinal (dysphagia: 12.9% in SMA, OR = 16, adjusted p-value<0.001); and skeletal (congenital anomalies of face and neck: 22.6% in SMA, OR = 5, adjusted p-value = 4E-2). Unsurprisingly, subjects in Group A also presented with early, but not definitive, signs of impending neuromuscular defects such as lack of coordination (67.7% in SMA, OR = 282, adjusted p-value<0.001), developmental delays and disorders (38.7% in SMA, OR = 39, adjusted p-value<0.001), and muscle weakness (19.4% in SMA, OR = 61, adjusted p-value<0.001).

Next, we analyzed phenotypes with significantly increased prevalence in Group B and again found non-neuromuscular phenotypes with significantly increased prevalence in SMA cases. These included: cardiovascular (cardiac shunt/ heart septal defects: 4.7% in SMA, OR = 5.8, adjusted p-value = 0.005; peripheral vascular disease, unspecified: 1.9% in SMA, OR = 122.7, adjusted p-value<0.001); gastrointestinal (GERD: 13.1% in SMA, OR = 4.1, adjusted p-value<0.001; constipation: 12.1% in SMA, OR = 2.8, adjusted p-value = 0.01); skeletal (kyphoscoliosis and scoliosis: 9.3% in SMA, OR = 8.7, adjusted p-value<0.001; congenital deformities of feet: 6.5% in SMA, OR = 11.2, adjusted p-value<0.001); and metabolic (symptoms concerning nutrition, metabolism, and development: 11.2% in SMA, OR 4.2, adjusted p-value = 3E-4). Similar to Group A, these subjects were also diagnosed with early signs of impending neuromuscular disorders such as abnormality of gait (12.1% in SMA, OR = 19.1, adjusted p-value<0.001) and lack of coordination (9.3% in SMA, OR = 16.1, adjusted p-value<0.001). Only acute pharyngitis (13.1% in SMA, OR = 0.4, adjusted p-value = 0.04) had lower prevalence in the SMA group.

Data within the pre-neuromuscular degeneration window for more mildly patients in Group C also revealed non-neuromuscular phenotypes including: cardiovascular (premature beats: 2.7% of SMA, OR = 3.3, adjusted p-value = 0.02); gastrointestinal (constipation: 7.1% of SMA, OR = 2.3, adjusted p-value = 0.006); skeletal (flat feet: 1.2% of SMA, OR = 5.0, adjusted p-value = 0.04); and sensorial (disturbance of skin sensation: 9.8% of SMA, OR = 2.3, adjusted p-value<0.001). Interestingly, in these more mildly affected patients who lived longer and survived to reproductive age, we observed phenotypes in their reproductive system (testicular hypofunction: 5.3% of SMA [9.5% of males], OR = 2.4, adjusted p-value = 0.02; infertility, male: 1.5% of SMA [2.6% of males], OR = 5.1, adjusted p-value = 0.01). As with Groups A and B, this cohort was also diagnosed with what are likely early, but commonly diagnosed, signs of neuromuscular degeneration (degeneration of intervertebral disc: 15.1% of SMA, OR = 2.7, adjusted p-value<0.001; spondylosis with myelopathy: 5.0% in SMA, OR = 11.7, adjusted p-value<0.001; abnormality of gait, 3.9% of SMA, OR = 2.7, adjusted p-value = 0.02).

### Temporal trajectories of the onset of categorized phenotypes

**Fig 5:**
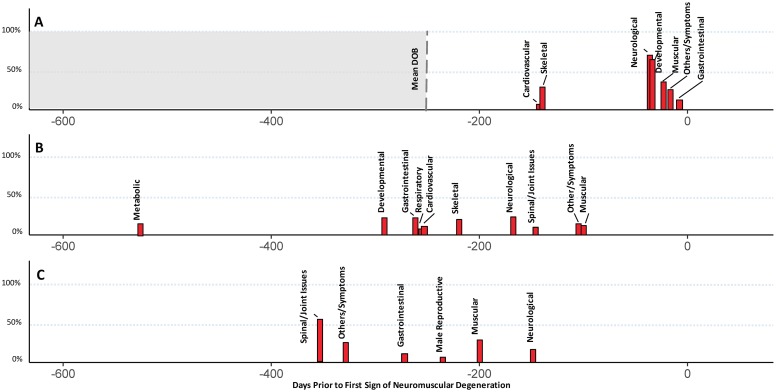
Temporal trajectories of grouped phenotypes. Data representing the median first day of onset for each physiological system (with >5% penetrance in SMA cohort) are presented with 0 representing a patient’s first sign of neuromuscular degeneration. The height of each rectangle represents prevalence. Data for each SMA cohort is presented separately: (A) Data from SMA patients in Group A (types I/II) with the mean date of birth (DOB) indicated; (B) Data from adolescent onset SMA patients in Group B (types IIIa/b); (C) Data from SMA patients in Group C (type IV).

We then grouped phenotypes from the pre-neuromuscular degeneration window with significant differences in rates of diagnoses between cohorts into categories representing independent physiological systems, as detailed in (S5–S7 Tables). In addition to running the logistic regression on these broader categories, we established a general time course for the appearance of these non-neuromuscular components of the disease. Timelines of phenotypes categorized by individual physiological system are presented along with odds ratios and prevalence in Table 3. Timelines for phenotypes with at least 5% prevalence were visualized based on median date of onset compared to the date of the first sign of neuromuscular involvement in Fig 5. These data establish that all types of SMA, even the most severe, can involve non-neuromuscular tissues in the period prior to significant neuromuscular decline and present a potential temporal ordering.

**Table 3:** Categorized differential phenotypes with timeline.

**Group A**:				
**Physiological System**	**OR**	**Ctrl Prev**	**SMA Prev**	**Median Days Before First Sign of Neuromuscular Degeneration**
Cardiovascular	42.23	0.2%	6.5%	-142
Skeletal	5.43	7.0%	29.0%	-141
Neurological	175.77	2.1%	71.0%	-38
Developmental	51.79	5.0%	64.5%	-36
Muscular	70.46	1.2%	35.5%	-23
Other/Symptoms	15.25	3.6%	25.8%	-17
Gastrointestinal	15.62	1.2%	12.9%	-8
**Group B**:				
**Physiological System**	**OR**	**Ctrl Prev**	**SMA Prev**	**Median Days Before First Sign of Neuromuscular Degeneration**
Metabolic	4.20	4.4%	15.0%	-526
Inflammation	9.91	0.2%	1.9%	-392
Developmental	11.76	2.9%	23.4%	-292
Skin	16.70	0.3%	3.7%	-291
Gastrointestinal	3.27	10.1%	23.4%	-262
Cardiovascular	11.28	1.3%	12.1%	-256
Respiratory	6.27	1.3%	7.5%	-254
Skeletal	10.21	3.7%	20.6%	-220
Neurological	9.50	6.3%	33.6%	-168
Spinal/Joint Issues	14.96	1.2%	10.3%	-147
Urinary	7.52	0.7%	3.7%	-134
Other/Symptoms	4.34	6.9%	15.0%	-105
Muscular	19.23	1.1%	12.1%	-101
**Group C**:				
**Physiological System**	**OR**	**Ctrl Prev**	**SMA Prev**	**Median Days Before First Sign of Neuromuscular Degeneration**
Spinal/Joint Issues	2.09	40.0%	55.8%	-354
Other/Symptoms	1.65	19.1%	24.6%	-329
Cardiovascular	3.32	0.8%	2.7%	-288
Gastrointestinal	2.41	6.0%	11.3%	-273
Skeletal	5.03	0.3%	1.2%	-243
Male_Reproductive	2.81	2.1%	6.8%	-236
Muscular	3.33	12.5%	28.5%	-201
Neurological	2.96	7.3%	16.9%	-149

Grouped phenotypes with increased prevalence for SMA patients by group are presented with Odds Ratios, prevalence in each cohort, and median days before the neuromuscular inflection point.

Data for Group A (Fig 5A) show that the earliest detected phenotypes were skeletal (-142 days, 6.5% in SMA, OR = 42.2) and cardiovascular (-141 days, 29.0% in SMA, OR = 5.4), while the remaining phenotypes—neurological (-38, 71% in SMA, OR = 175.8), failure to achieve developmental milestones (-36 days, 64.5% in SMA, OR = 51.8); and muscular (-23 days, 35.5% in SMA, OR = 70.5)—were found closer to the first signs of neuromuscular degeneration. Data for Group B (Fig 5B) show that metabolic defects (-526 days, 15% in SMA, OR = 4.2) were the earliest detectable phenotypes, followed by developmental (-292 days, 23.4% in SMA, OR = 11.8), gastrointestinal (-262 days, 23.4% in SMA, OR = 3.3), cardiovascular (-256 days, 12.1% in SMA, OR = 11.3), respiratory (-254 days, 7.5% in SMA, OR = 6.3), and skeletal (-220 days, 20.6% in SMA, OR = 10.2) phenotypes. These patients were typically diagnosed with neurological (-168 days, 33.6% in SMA, OR = 9.5), spinal/joint issues (-147 days, 10.3% in SMA, OR = 15.0) and musculoskeletal (-101 days, 12.1% in SMA, OR = 19.2) phenotypes only within the last 6 months before their first sign of neuromuscular dysfunction. Data from Group C (Fig 5C) highlight that the earliest phenotypes represented spinal/joint issues (-354 days, 55.8% in SMA, OR = 2.1), followed by gastrointestinal (-273 days, 11.3% in SMA, OR = 2.4) and male reproductive (-236 days, 6.8% in SMA [11.8% in males], OR = 2.81) issues. Muscular (-201 days, 28.5% in SMA, OR = 3.3) and neurological (-149 days, 16.9% in SMA, OR = 3.0) phenotypes were, again, the last to be diagnosed, being noted around 6 months prior to the first sign of neuromuscular involvement. The R^2^ coefficients of correlation for these results with enrollment duration were 0.21, 0.10, and 0.19 for Groups A, B, and C, respectively, indicating little of the variation can be explained by enrollment duration.

## Discussion

Growing evidence indicates that SMA is not solely a motor neuron disease. This includes observations made using a severe SMA mouse model, post-SMA diagnosis clinical studies and reports based on small numbers of patients. This is consistent with gene expression data showing that SMN is transcribed in all tissues and cellular data demonstrating that SMN plays a broad and important role in mRNA splicing in all cells. However, evidence is lacking that SMN deficiency directly translates into detectable non-neuromuscular phenotypes in humans, and, if so, at which times during disease progression. One reason for this is that historical SMA studies focused on patients after SMA diagnosis [33–38], when phenotypes can be masked by neuromuscular degeneration or characterized as downstream complications. Given the current and pending approvals of SMN increasing therapies, understanding the complete pathophysiology of SMA is of critical importance in order to provide more comprehensive measures of treatment efficacy, support comparative effectiveness studies of local versus systemic interventions, and to determine whether early clinically detectable signs exist that could be used to support pre-symptomatic intervention in milder patients.

In this study, we first revealed a broad spectrum of phenotypes that were differentially diagnosed in the SMA population compared to controls during the entire coverage window, including pre-and post-SMA diagnosis. We then focused on investigating SMA patient health prior to the first clinical sign of neuromuscular degeneration, when the cause of non-neuromuscular phenotypes is more likely to be independently caused by SMN deficiency. To our knowledge, this is the first large-scale study investigating pre-diagnostic SMA disease progression. Our study revealed numerous non-neuromuscular phenotypes in patients with varying severities of disease. Notably, peripheral phenotypes including those in the cardiovascular, gastrointestinal, metabolic, reproductive, and skeletal systems were detectable prior to any sign of neurological or muscular defects.

Some of the phenotypes we detected in SMA patients have been reported in SMA mouse models despite their short survival time. For example, SMA mice have been demonstrated to have vascular defects that lead to necrosis of the tail and ears [39, 40]. Our results indicate that SMA patients are also more likely to be diagnosed with vascular defects, such as peripheral vascular disease, chronic venous insufficiency, and chronic vascular insufficiency of the intestines. SMA mouse models have been reported to present with cardiac failure, remodeling, and septal defects [41–43], and our study found evidence of valve disorders, cardiomyopathies, septal defects, and premature beats spanning all three SMA severity Groups. Additionally, SMA model mice have reduced numbers of intestinal villi that are blunt and club-shaped with severe intramural edema [44]. This may correlate with our finding that all three Groups of SMA patients have diagnoses of gastrointestinal disorders and dysfunction. However, gastrointestinal phenotypes, such as dysphagia and constipation, diagnosed in patients could be a consequence of early muscle weakness. One of the more surprising findings of our study is dysfunction in the male reproductive system of adult-onset SMA patients in Group C. This is not completely unprecedented, as it aligns with recent studies in an SMA mouse model showing infertility in males and developmental issues in their testes [7, 8] and with an anecdotal human study of two subjects with atrophic testes [45]. If male reproductive dysfunction is confirmed to be a factor in this disease, then hormone markers such as testosterone may prove to function as biomarkers of disease severity that track with therapeutic efficacy.

Our findings indicate that it may be possible to identify potential patients for genetic testing before major neurological damage more effectively. For example, symptoms that could motivate genetic testing for SMA in infants or adolescents may include a history of diagnoses for cardiovascular disorders, gastrointestinal problems, or skeletal deformities when a child fails to meet traditional developmental milestones. For the milder cases, genetic testing could be motivated through a combination of more common defects such as skeletal deformities (e.g., flat feet or scoliosis), constipation, disturbances in skin sensation, or irregular cardiovascular function occurring prior to common early signs of neurological issues such as spondylosis or degeneration of intervertebral disks. In men, another early sign could include genitourinary issues such as testicular hypofunction. However, these findings need validation by studies with different subjects and determination of whether sets of diagnoses translate into sufficient conversion rates to be considered actionable. Finally, it would also be valuable to run a prospective study of pre-symptomatic patients to gather information on the entire set of tissues that could be affected in particular individuals, as our results are based on more routine tests not developed for exploring systemic nature of a disease. It may be that it is an unusual combination of affected tissues that most effectively tracks pre-neuromuscular disease progression of later onset of SMA.

More broadly, our results indicate that SMA is not a pure motor neuron disease, since other tissues are implicated. Motor neuron dysfunction may not even be the earliest clinically detectable manifestation of SMN deficiency even though this dysfunction ultimately is clinically of most significance, particularly in children with early-onset SMA. Application of these findings may include the development of broader endpoints for SMA clinical trials, a framework for comparing efficacy of systemic versus local SMN increasing therapeutics and the provision of a multi-systems framework that can guide the search for non-neuromuscular biomarkers that may track patient disease before and during treatment more dynamically. In that regard, SMA may resemble other disorders involving changes in CNS tissue, such as Parkinson’s disease, that are well-known to have extra-CNS symptomology [46]. Most importantly, studies like ours could highlight specific components of SMA that might require more targeted therapies.

There are limitations and potential pitfalls to our study. The first is that our findings are built on health insurance claims data, which were generated for billing purposes and not for research. However, it has been shown that insurance claims are equivalently accurate to electronic health records, a data source widely used in medical research [47]. Furthermore, the fact that healthcare is not universal in the United States, where the data was generated, may lead to biases in our findings. A recent review of healthcare coverage in the United States revealed that the majority of individuals are covered through commercial providers, with employer-based coverage being the most common, followed by Medicaid and Medicare. They further showed a difference in uninsured rates by race with non-Hispanic Whites being the most insured and Hispanics being the least [48]. A second potential pitfall is that many phenotypes, especially those not directly linked to billable events, are likely to have gone undiagnosed, thereby hindering our ability to properly identify neuromuscular involvement and also reducing the detected prevalence of other phenotypes. One potential consequence is that some patients had been previously diagnosed with SMA prior to their coverage window. However, the required six-month window prior to detectable neuromuscular degeneration reduces the risk that we are missing a subject’s first SMA diagnosis. Another potential challenge in our data interpretation is that, despite the alignment with mouse data, our non-neuromuscular findings could still be secondary to the underlying neurological degeneration that may not have been symptomatic at that point. Finally, while this study is the largest SMA study to date and the only one focusing on subject health prior at points prior to SMA diagnosis, our numbers did not enable us to control for all possible confounding variables. Even considering these potential limitations, we are confident in the results and hope they may motivate larger studies to validate and extend our findings in other pre-symptomatic cohorts.

In conclusion, this work presents a phenome-wide analysis of SMA progression, demonstrating its association with a range of neuromuscular and non-neuromuscular phenotypes. Given the extended lifespan of patients treated by SMN increasing therapeutics, our data may provide insights into other physiological systems that will need dedicated care. Our temporal analysis indicates that many non-neuromuscular phenotypes are present prior to early manifestations of neuromuscular degeneration. This points not only to a primary relationship of these symptoms with SMN deficiency, but also towards the possibility of their use in predicting time to neuromuscular symptom onset for treatment initiation prior to irreversible nervous system damage in later stage patients who may not have been screened at birth or who want to delay treatment while they remain asymptomatic.

## Supporting information

S1 Table Details of ICD9 codes used. List of the diagnosis codes used as inclusion and exclusion. criteria used for patient selection and establishment of critical time points in disease progression. These diagnosis codes include: SMA codes; any pregnancy codes or codes related to pregnancy complications; and codes related to any neuromuscular disease (MD, SMA, etc.) or evidence of major neuromuscular degeneration used to determine the neuromuscular inflection point.
(XLSX)

S2 Table Differential PheWAS codes for Group A from entire coverage analysis. PheWAS codes representing differential diagnoses from the entire coverage analysis for SMA patients in Group A are presented. Data includes OR, prevalence in each cohort, and adjusted p-value.
(XLSX)

S3 Table Differential PheWAS codes for Group B from entire coverage analysis. PheWAS codes representing differential diagnoses from the entire coverage analysis for SMA patients in Group B are presented. Data includes OR, prevalence in each cohort, and adjusted p-value.
(XLSX)

S4 Table Differential PheWAS codes for Group C from entire coverage analysis. PheWAS codes representing differential diagnoses from the entire coverage analysis for SMA patients in Group C are presented. Data includes OR, prevalence in each cohort, and adjusted p-value.
(XLSX)

S5 Table Differential PheWAS codes for Group A from pre-neuromuscular degeneration. PheWAS codes representing differential diagnoses from the pre-neuromuscular degeneration analysis for SMA patients in Group A are presented. Data includes OR, prevalence in each cohort, and adjusted p-value.
(XLSX)

S6 Table Differential PheWAS codes for Group B from pre-neuromuscular degeneration. PheWAS codes representing differential diagnoses from the pre-neuromuscular degeneration analysis for SMA patients in Group B are presented. Data includes OR, prevalence in each cohort, and adjusted p-value.
(XLSX)

S7 Table Differential PheWAS codes for Group C from pre-neuromuscular degeneration. PheWAS codes representing differential diagnoses from the pre-neuromuscular degeneration analysis for SMA patients in Group C are presented. Data includes OR, prevalence in each cohort, and adjusted p-value.
(XLSX)

## References

[1] Kolb S.J. and Kissel J.T., Spinal muscular atrophy: a timely review. Arch Neurol, 2011 68(8): p. 979–84. 10.1001/archneurol.2011.74 21482919 PMC3860273

[2] Lefebvre S., Burlet P., Viollet L., Bertrandy S., Huber C., Belser C. et al, A novel association of the SMN protein with two major non-ribosomal nucleolar proteins and its implication in spinal muscular atrophy. Hum Mol Genet, 2002 11(9): p. 1017–27. 11978761 10.1093/hmg/11.9.1017

[3] Swaiman, K.F., Ashwal, S. Ferriero, D.M., Schor, N.F., Finkel R.S., Gropman A.L., et al, (Darras BT, Monani UR, De Vivo DC. Genetic disorders affecting the motor neuron: spinal muscular atrophy. Chapter 139.) In: Swaiman’s Pediatric Neurology E-Book: Principles and Practice. 2017: Elsevier Health Sciences.

[4] Hamilton G. and Gillingwater T.H., Spinal muscular atrophy: going beyond the motor neuron. Trends Mol Med, 2013 19(1): p. 40–50. 10.1016/j.molmed.2012.11.002 23228902

[5] Shababi M., Lorson C.L. and Rudnik-Schöneborn S.S., Spinal muscular atrophy: a motor neuron disorder or a multi-organ disease? J Anat, 2014 224(1): p. 15–28. 10.1111/joa.12083 23876144 PMC3867883

[6] Ng S.Y., Soh B.S., Rodriguez-Muela N., Hendrickson D.G., Price F., Rinn J.L., et al, Genome-wide RNA-Seq of Human Motor Neurons Implicates Selective ER Stress Activation in Spinal Muscular Atrophy. Cell Stem Cell, 2015 17(5): p. 569–84. 10.1016/j.stem.2015.08.003 26321202 PMC4839185

[7] Riessland M., Ackermann B., Förster A., Jakubik M., Hauke J., Garbes L., et al, SAHA ameliorates the SMA phenotype in two mouse models for spinal muscular atrophy. Hum Mol Genet, 2010 19(8): p. 1492–506. 10.1093/hmg/ddq023 20097677

[8] Ottesen E.W., Howell M.D., Singh N.N., Seo J., Whitley E.M. and Singh R.N., Severe impairment of male reproductive organ development in a low SMN expressing mouse model of spinal muscular atrophy. Sci Rep, 2016 6: p. 20193 10.1038/srep20193 26830971 PMC4735745

[9] Bowerman M., Swoboda K.J., Michalski J.P., Wang G.S., Reeks C., Beauvais A., et al, Glucose metabolism and pancreatic defects in spinal muscular atrophy. Ann Neurol, 2012 72(2): p. 256–68. 10.1002/ana.23582 22926856 PMC4334584

[10] The Genotype-Tissue Expression (GTEx) project. Nat Genet, 2013 45(6): p. 580–5. 10.1038/ng.2653 23715323 PMC4010069

[11] Groen E.J., Perenthaler E., Courtney N.L., Jordan C.Y., Shorrock H.K., Van Der Hoorn D., et al, Temporal and tissue-specific variability of SMN protein levels in mouse models of spinal muscular atrophy. Hum Mol Genet, 2018. 10.1093/hmg/ddy195 PMC6077828 29790918

[12] Gubitz A.K., Feng W., and Dreyfuss G., The SMN complex. Exp Cell Res, 2004 296(1): p. 51–6. 10.1016/j.yexcr.2004.03.022 15120993

[13] Kolb S.J., Battle D.J., and Dreyfuss G., Molecular functions of the SMN complex. J Child Neurol, 2007 22(8): p. 990–4. 10.1177/0883073807305666 17761654

[14] Singh R.N., Howell M.D., Ottesen E.W. and Singh N.N., Diverse role of survival motor neuron protein. Biochim Biophys Acta, 2017 1860(3): p. 299–315. 10.1016/j.bbagrm.2016.12.008 PMC5325804 28095296

[15] Le T.T., Pham L.T., Butchbach M.E., Zhang H.L., Monani U.R., Coovert D.D., et al, SMNΔ7, the major product of the centromeric survival motor neuron (SMN2) gene, extends survival in mice with spinal muscular atrophy and associates with full-length SMN. Human molecular genetics, 2005 14(6): p. 845–857. 10.1093/hmg/ddi078 15703193

[16] Bebee T.W., Dominguez C.E., and Chandler D.S., Mouse models of SMA: tools for disease characterization and therapeutic development. Hum Genet, 2012 131(8): p. 1277–93. 10.1007/s00439-012-1171-5 22543872

[17] Finkel R.S., Chiriboga C.A., Vajsar J., Day J.W., Montes J., De Vivo D.C., et al Treatment of infantile-onset spinal muscular atrophy with nusinersen: a phase 2, open-label, dose-escalation study. The Lancet, 2016 388(10063): pp.3017–3026. 10.1016/S0140-6736(16)31408-8 27939059

[18] Scoto M., Finkel R.S., Mercuri E. and Muntoni F., Therapeutic approaches for spinal muscular atrophy (SMA). Gene Ther, 2017 24(9): p. 514–519. 10.1038/gt.2017.45 28561813

[19] Paez-Colasante X., Seaberg B., Martinez T.L., Kong L., Sumner C.J. and Rimer M., Improvement of neuromuscular synaptic phenotypes without enhanced survival and motor function in severe spinal muscular atrophy mice selectively rescued in motor neurons. PLoS One, 2013 8(9): p. e75866 10.1371/journal.pone.0075866 24086650 PMC3781079

[20] Lee A.J., Awano T., Park G.H. and Monani U.R., Limited phenotypic effects of selectively augmenting the SMN protein in the neurons of a mouse model of severe spinal muscular atrophy. PLoS One, 2012 7(9): p. e46353 10.1371/journal.pone.0046353 23029491 PMC3459898

[21] Wishart T.M., Mutsaers C.A., Riessland M., Reimer M.M., Hunter G., Hannam M.L., Dysregulation of ubiquitin homeostasis and beta-catenin signaling promote spinal muscular atrophy. J Clin Invest, 2014 124(4): p. 1821–34. 10.1172/JCI71318 24590288 PMC3973095

[22] Hua Y., Liu Y.H., Sahashi K., Rigo F., Bennett C.F. and Krainer A.R., Motor neuron cell-nonautonomous rescue of spinal muscular atrophy phenotypes in mild and severe transgenic mouse models. Genes Dev, 2015 29(3): p. 288–97. 10.1101/gad.256644.114 25583329 PMC4318145

[23] Zhou H., Meng J., Marrosu E., Janghra N., Morgan J. and Muntoni F., Repeated low doses of morpholino antisense oligomer: an intermediate mouse model of spinal muscular atrophy to explore the window of therapeutic response. Hum Mol Genet, 2015 24(22): p. 6265–77. 10.1093/hmg/ddv329 26264577 PMC4614699

[24] Tizzano E.F. and Finkel R.S., Spinal muscular atrophy: A changing phenotype beyond the clinical trials. Neuromuscul Disord, 2017 27(10): p. 883–889. 10.1016/j.nmd.2017.05.011 28757001

[25] Wirth B., Barkats M., Martinat C., Sendtner M. and Gillingwater T.H., Moving towards treatments for spinal muscular atrophy: hopes and limits. Expert Opin Emerg Drugs, 2015 20(3): p. 353–6. 10.1517/14728214.2015.1041375 25920617

[26] Luo J., Wu M., Gopukumar D. and Zhao Y., Big Data Application in Biomedical Research and Health Care: A Literature Review. Biomed Inform Insights, 2016 8: p. 1–10. 10.4137/BII.S31559 PMC4720168 26843812

[27] Goldstein B.A., Navar A.M., Pencina M.J. and Ioannidis J., Opportunities and challenges in developing risk prediction models with electronic health records data: a systematic review. J Am Med Inform Assoc, 2017 24(1): p. 198–208. 10.1093/jamia/ocw042 27189013 PMC5201180

[28] Miotto R., Wang F., Wang S., Jiang X. and Dudley J.T., Deep learning for healthcare: review, opportunities and challenges. Brief Bioinform, 2017. 10.1093/bib/bbx044 PMC6455466 28481991

[29] Armstrong E.P., Malone D.C., Yeh W.S., Dahl G.J., Lee R.L. and Sicignano N., The economic burden of spinal muscular atrophy. J Med Econ, 2016 19(8): p. 822–6. 10.1080/13696998.2016.1198355 27264163

[30] Denny J.C., Bastarache L., Ritchie M.D., Carroll R.J., Zink R., Mosley J.D., et al, Systematic comparison of phenome-wide association study of electronic medical record data and genome-wide association study data. Nat Biotechnol, 2013 31(12): p. 1102–10. 10.1038/nbt.2749 24270849 PMC3969265

[31] Team, R.C. R: A language and environment for statistical computing. 2015; https://www.r-project.org/.

[32] Benjamini Y. and Hochberg Y., Controlling the False Discovery Rate: A Practical and Powerful Approach to Multiple Testing. Journal of the Royal Statistical Society. Series B (Methodological), 1995 57(1): p. 289–300.

[33] Zerres K. and Rudnik-Schoneborn S., Natural history in proximal spinal muscular atrophy. Clinical analysis of 445 patients and suggestions for a modification of existing classifications. Arch Neurol, 1995 52(5): p. 518–23. 7733848 10.1001/archneur.1995.00540290108025

[34] Zerres K., Rudnik-Schöneborn S., Forrest E., Lusakowska A., Borkowska J. and Hausmanowa-Petrusewicz I., A collaborative study on the natural history of childhood and juvenile onset proximal spinal muscular atrophy (type II and III SMA): 569 patients. J Neurol Sci, 1997 146(1): p. 67–72. 9077498 10.1016/s0022-510x(96)00284-5

[35] Kaufmann P., McDermott M.P., Darras B.T., Finkel R.S., Sproule D.M., Kang P.B., et al, Prospective cohort study of spinal muscular atrophy types 2 and 3. Neurology, 2012 79(18): p. 1889–97. 10.1212/WNL.0b013e318271f7e4 23077013 PMC3525313

[36] Bertini E. and Mercuri E., Motor neuron disease: A prospective natural history study of type 1 spinal muscular atrophy. Nat Rev Neurol, 2018 14(4): p. 197–198. 10.1038/nrneurol.2017.189 29348544

[37] Kaufmann P., McDermott M.P., Darras B.T., Finkel R.S., Sproule D.M., Kang P.B., et al, Evaluation of SMN protein, transcript, and copy number in the biomarkers for spinal muscular atrophy (BforSMA) clinical study. PLoS One, 2012 7(4): p. e33572 10.1371/journal.pone.0033572 22558076 PMC3338744

[38] Finkel R.S., Crawford T.O., Swoboda K.J., Kaufmann P., Juhasz P., Li X., et al, Candidate proteins, metabolites and transcripts in the Biomarkers for Spinal Muscular Atrophy (BforSMA) clinical study. PLoS One, 2012 7(4): p. e35462 10.1371/journal.pone.0035462 22558154 PMC3338723

[39] Hsieh-Li H.M., Chang J.G., Jong Y.J., Wu M.H., Wang N.M., Tsai C.H., et al, A mouse model for spinal muscular atrophy. Nat Genet, 2000 24(1): p. 66–70. 10.1038/71709 10615130

[40] Narver H.L., Kong L., Burnett B.G., Choe D.W., Bosch-Marcé M., Taye A.A., et al, Sustained improvement of spinal muscular atrophy mice treated with trichostatin A plus nutrition. Ann Neurol, 2008 64(4): p. 465–70. 10.1002/ana.21449 18661558 PMC10103738

[41] Bevan A.K., Hutchinson K.R., Foust K.D., Braun L., McGovern V.L., Schmelzer L., et al, Early heart failure in the SMNDelta7 model of spinal muscular atrophy and correction by postnatal scAAV9-SMN delivery. Hum Mol Genet, 2010 19(20): p. 3895–905. 20639395 10.1093/hmg/ddq300 PMC2947399

[42] Heier C.R., Satta R., Lutz C. and DiDonato C.J., Arrhythmia and cardiac defects are a feature of spinal muscular atrophy model mice. Hum Mol Genet, 2010 19(20): p. 3906–18. 10.1093/hmg/ddq330 20693262 PMC2947406

[43] Shababi M., Habibi J., Yang H.T., Vale S.M., Sewell W.A. and Lorson C.L., Cardiac defects contribute to the pathology of spinal muscular atrophy models. Hum Mol Genet, 2010 19(20): p. 4059–71. 10.1093/hmg/ddq329 20696672

[44] Schreml J., Riessland M., Paterno M., Garbes L., Roßbach K., Ackermann B., et al, Severe SMA mice show organ impairment that cannot be rescued by therapy with the HDACi JNJ-26481585. Eur J Hum Genet, 2013 21(6): p. 643–52. 10.1038/ejhg.2012.222 23073311 PMC3658191

[45] Richert J.R., Antel J.P., Canary J.J., Maxted W.C. and Groothuis D., Adult onset spinal muscular atrophy with atrophic testes: report of two cases. J Neurol Neurosurg Psychiatry, 1986 49(5): p. 606–8. 10.1136/jnnp.49.5.606-a PMC1028824 3711928

[46] Johnson M.E., Labrie V., Brundin L. and Brundin P., Triggers, Facilitators, and Aggravators: Redefining Parkinson’s Disease Pathogenesis. Trends Neurosci, 2018. 10.1016/j.tins.2018.09.007 PMC6623978 30342839

[47] Kottke T.E., Baechler C.J. and Parker E.D., Accuracy of heart disease prevalence estimated from claims data compared with an electronic health record. Prev Chronic Dis, 2012 9: p. E141 10.5888/pcd9.120009 22916996 PMC3475521

[48] Berchick E.R., Hood E. and Barnett J.C., Health Insurance Coverage in the United States: 2017 Current Population Reports. US Government Printing Office, Washington, DC 2018 p. 60–264.

